# Body distribution of ^11^C-methionine and ^18^FDG in rat measured by microPET

**DOI:** 10.2478/v10102-011-0010-1

**Published:** 2011-03

**Authors:** Svorad Štolc, Lucia Jakubíková, Ivica Kukurová

**Affiliations:** Biont, Inc., Bratislava, Slovak Republic

**Keywords:** ^11^C-methionine, ^18^FDG, biodistribution, microPET, rat

## Abstract

Compounds ^18^F-fluorodeoxyglucose (^18^FDG) and ^11^C-methionine (^11^C-MET) are radiodiagnostics frequently used in clinical Positron Emission Tomography (PET) as well in preclinical studies of various pathologies. The present study was focused on the comparison of biodistribution of both radiotracers in intact Wistar rats. The animals were scanned by microPET twice. The first scanning was done after ^11^C-MET administration, the second scan followed 5–7 days later using ^18^FDG. The radiotracers were injected into the tail vein of animals in isoflurane anesthesia. After a redistribution period, whole body scans were obtained using eXplore Vista SrT GE tomograph. Accumulation of the drugs in tissues was expressed in relative values (% ID/g) in selected regions of interest. As arbitrary reference tissue for drug accumulation, the sternoclavicular area was used. ^18^C-MET was found remarkably cumulating especially in the liver, spleen and distal part of the gastrointestinal tract. The compound was accumulated in the liver 6.9±0.92 (mean±SEM) times more intensively than in the reference tissue. The respective value for spleen and cecum/colon was 5.62±0.81 and 3.56±0.14 times. Accumulation of ^11^C-MET in other body parts including the brain and heart was very low and was apparently equal to the arbitrary tissue (0.13±0.01% ID/g). In the same animals ^18^FDG (biontFDG) was remarkably cumulated especially in Harderian glands compared to arbitrary tissue background (11.02±1.00 times), heart (7.52±1.70 times), brain (6.14±0.37 times), and colon (5.68±0.31 times). ^18^FDG accumulation in the liver, spleen and other organs was apparently not different from that found in the background (0.14±0.02% ID/g). The data obtained may serve as reference values in further microPET preclinical studies with ^11^C-MET and ^18^FDG under the given conditions.

## Introduction

Positron emission tomography is used as valuable diagnostic technique in numerous pathologies (Phelps 2000, 2004). ^18^F-flurodeoxyglucose (^18^FDG) and ^11^C-methionine (^11^C-MET) are radiodiagnostics frequently used in this technique. They enter metabolic processes at different sites, hence their body distribution as well as affinities to different tissues are also different. ^18^FDG easily enters cells by the activity of glucose pumps along with glucose. Accumulation of this compound in tissues is considered to be a measure of energetic metabolism in cells (Wahl, 1996; Pauwels *et al*., 1998; Chen & Dehdashti, 2005). Methionine is transported into cells by a complex system of transporters (Singhal *et al*., 2008). It participates in several metabolic pathways, mainly in proteosynthesis, methylation, polyamine generation, transsulfuration (Finkelstein & Martin, 1984). However, acute accumulation of ^11^C-MET in tissues is linked mostly to the rate of proteosynthesis (Ishiwata *et al*., 1988; Fischman *et al*., 1998). Besides their use in clinical practice both drugs may be also used in preclinical studies focusing on monitoring various body functions. However, direct comparison of body distribution of the two compounds in the normal rat is not available in the literature. Hence, the aim of the study was to compare whole body distribution of ^11^C-MET and ^18^FDG after IV administration to intact rats.

## Methods

Male Wistar rats (n=4) weighing 192–217 g were used in the study. The animals were kept under standard conditions. They were deprived of food with free access to water 12 hours prior to experiment. ^11^C-MET and ^18^FDG were synthesized in house by Cyclone 18/9 cyclotron.

Rats were anesthetized with isoflurane/oxygen mixture (5% induction and 1.5% for maintenance) and ^11^C-MET with activity of approximately 50 MBq in 0.05–0.5 ml of buffered saline was injected into the tail vein. After 15 min of tracer distribution in the body, the animals were placed in the prone position (with head first) on the thermostatically heated bed of the microPET scanner (eXplore VISTA SrT GE) and scanning started. As field of view (FOV) of the scanner is 20 mm in axial direction, for whole body image the animals were scanned in sequence of 8–10 horizontally moved bed positions, depending on the size of the rat. Each bed position was scanned for 10 minutes, thus scanning time was 80 to 100 minutes. Activities measured at different time intervals were automatically corrected by the scanner for radionuclide decay. After five to seven days, the whole procedure was repeated with ^18^FDG IV injected, allowing 60 min for the distribution of the tracer in the body before measurement.

The shorter distribution time used with ^11^C-MET compared to that with ^18^FDG was necessitated by shorter decay with half-time in ^11^C than ^18^F (t_1/2_=20.4 and 109.8 min, respectively).

After radioactivity of administered ^18^FDG decreased sufficiently (>10 half-times), the animals were sacrificed and autopsy was carried out, focused on position of individual organs within the body.

### Analysis of PET data

Images were reconstructed and analyzed by eXplore VISTA 3.1 Analysis & Visualisation software. For image reconstruction 3D FORE/2D OSEM algorithms were used. Regions of interest (ROI) with evident tracer accumulation were identified and manually marked in the area of the brain, heart, liver, spleen, kidneys, colon, and Harderian glands. Sternoclavicular area was considered to be arbitrary tissue background. The ROI dimensions were at least 2.5 times resolution of the camera which is 1.5 mm. Relative accumulation of the radioactivity in particular ROI was expressed as% ID/g.

Statistical analysis was done in GraphPad Prism software. The data are expressed as means with their standard errors (SEM).

### Calculations

Distribution of the radiodiagnostics administered was expressed by percentage of the radioactivity administered to the animal relative to one gram of the tissue selected (%D/g). The variable was calculated in each selected region of interest (ROI) according to Gambhir (2004), Shimoji *et al*. (2004), Aliaga *et al*. (2007) by the formula:

(1) $$\%\text{ID}/\text{g}=100\times \frac{\text{c}[\text{kBq}/\text{c}{\text{m}}^{3}]}{\text{TD}[\text{kBq}]}/(\rho [\text{g}/\text{c}{\text{m}}^{3}])$$

where *c* is radionuclide concentration in ROI expressed by the formula (2), *ρ* is tissue density (*i.e.* ~1 g/cm^3^), TD is total injected dose corrected to scan onset time.

(2) $$\text{c}=\text{C}\times \frac{\text{cps in a animal ROI}[\text{cps}]}{\text{ROI volume in image}[\text{c}{\text{m}}^{\text{3}}]}$$

where C is calibration factor calculated using calibration phantom (cylinder).

(3) $$\text{C}=\frac{\text{radionuclide concentration in cylinder}[\text{kBq}/\text{c}{\text{m}}^{3}]}{\frac{\text{cps in a ROI}}{\text{ROI volume in image}}[\text{cps}/\text{c}{\text{m}}^{3}]}$$

## Results

**Figure 1:**
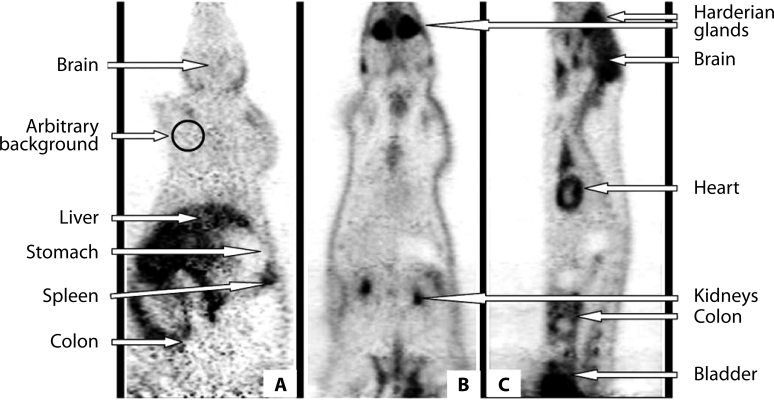
Accummulation of the radiotracers in the rat. A – ^11^C-MET, coronal view, B and C – ^18^FDG, coronal and sagittal view, respectively.

Accumulation of ^11^C-MET and ^18^FDG following IV administration was measured in whole body of animals after chosen distribution periods. Static measurement lasting 10 min for each 20 mm section of the body was used. In Figure 1 an example of body scans of one of the animals is presented after IV administration of ^11^C-MET (panel A) and ^18^FGD (panels Band C). Identification of the organs assessed was facilitated using 3D reconstruction of the scans with rotation of the scanned objects as well as by anatomical dissection made *post mortem*.

**Figure 2:**
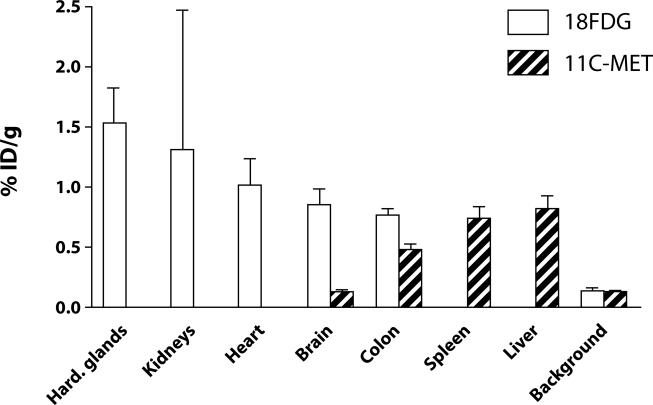
Accumulation of ^11^C-MET and ^18^FDG administered IV in various organs expressed as percentage of the injected dose per gram of the tissue (% ID/g). The sternoclavicular area was selected as abitrary reference background. Means with SEM are shown.

Accumulation of the compounds expressed in relative values as% ID/g in selected organs are presented in Figure 2. The highest accumulation of ^11^C-MET was observed in the liver followed by the spleen and colon. Compared to that in the arbitrary reference area, accumulation ranged from >6 times higher in the liver to 3.5 times higher in the colon (Table 1). The compound was apparently not accumulating in the brain in the given time period.

**Table 1:** Ratio of the % ID/g values in some organs to that in the arbitrary background following ^11^C-MET IV administration (n=4).

	%ID/g in organ/background					
			
organ	animal 1	animal 2	animal 3	animal 4	average	SEM
**liver**	5.87	5.27	9.00	5.00	6.29	0.92
**spleen**	4.67	4.36	7.92	5.53	5.62	0.81
**colon**	3.93	3.27	3.50	3.53	3.56	0.14
**brain**	0.80	0.73	1.33	1.07	0.98	0.14

^18^FDG revealed a different accumulation profile and intensity than ^11^C-MET (Figure 2). It was accumulated most markedly in Harderian glands with as high as 11 times more intensively captured here than in the reference region (Table 2). Accumulation in the kidney, heart, brain, and colon was gradually lower, being in the colon still more than 5.6 times higher than in the reference area. Data on ^18^FDG accumulation in the kidney obtained in the present study are not reliable because of the low number of measurements (n=3) and their high variability. Consequently, any statement on ^18^FDG accumulation in this tissue has only limited reliability.

**Table 2:** Ratio of the % ID/g values in some organs to that in the arbitrary background following ^18^FDG IV administration (n=3).

	% ID/g in organ/background				
			
organ	animal 1	animal 2	animal 3	average	SEM
**Hard. glands**	9.08	11.58	12.41	11.02	1.00
**kidneys**	1.00	30.33	1.00	10.78	9.78
**heart**	4.83	10.67	7.06	7.52	1.70
**brain**	5.42	6.42	6.59	6.14	0.37
**colon**	6.17	5.75	5.12	5.68	0.31

## Discussion

The present study revealed different profiles of body distribution of ^11^C-MET and ^18^FDG in the intact rat. Especially a comparatively high uptake of ^11^C-MET was observed in the liver, spleen, and colon and an apparently very low uptake occurred in the brain and other organs. On the other hand, a high uptake of ^18^FDG was found in the Harderian glands, heart, brain and colon.

Several authors studied methionine body distribution in various objects. Edwards *et al*. (1963) analyzed the fate of ^14^C-L-methionine in numerous tissues 30 min after its administration to Wistar rats. They observed the following profile – pancreas>liver>bone marrow>kidneys>lungs. They concluded that the liver plays an important role in the metabolismof methionine. Accordingly, Kubota *et al*. (1984) found a higher accumulation of ^11^C-MET in the pancreas than in the liver in the rat with hepatoma AH109A. Amano *et al*. (1998) studied accumulation of ^11^C-L-methionine in nude mice with human breast carcinoma MCF-7. The most remarkable uptake of the tracer was found in the liver. The uptake was decreasing in the following order: liver > kidney > spleen > stomach > blood > skeletal muscle. Cook *et al*. (1999) in their review article about clinical PET applications and investigations indicated low uptake in the central nervous system and high uptake in the abdomen and pelvis. They described high liver activity, variable accumulation in bowel and bladder and low activity in the kidney.

Our observations of high accumulation of ^11^C-MET in the liver and spleen are in agreement with the above findings. However, no evident uptake in kidneys and lungs was observed, unlike that found by Edwards *et al*. (1963) and Amano *et al*. (1998). The differences might be related to different ways of administration of the radiodiagnostics, by interspecies differences, and/or by different health status of the animals. Moreover, microPET itself without corresponding imaging techniques (MRI, CT, autoradiography) did not allow to draw reliable conclusions of ^11^C-MET accumulation in smaller organs in the present study.

Low basal uptake of ^11^C-MET observed in the brain and heart allows to expect facilitated detection of pathologies causing enhanced tissue uptake of methionine in these organs induced by increased proteosynthesis (tumors, inflammation, tissue reparation processes, etc.).

Data on body distribution of ^18^FDG were presented by numerous authors. Among them Cook *et al*. (1999) summarized normal distribution of ^18^FDG in men. The highest uptake was typically in the central nervous system, variable uptake was found in the cardiovascular and gastrointestinal system and low grade accumulation was in the liver, spleen, skeletal muscle and lung. Yamaguchi *et al*. (1999) described distribution of ^18^FDG in men after PO administration. They observed organ accumulation in the following order: intestine > brain > liver > stomach. The high accumulation in the intestine was not surprising due to the way of administration of the tracer. In PET Protocol for Oncological Diagnostics of the Munich University (Anonym, 2000), the highest ^18^FDG accumulation was found in the brain followed by that in the liver and mediastinum. Enhanced ^18^FDG uptake was also found in malignant tumors. Tan & Ong (2004) reported ^18^FDG uptake in men: cerebellum > myocardium > tonsiles > liver > spleen > stomach > rectum.

Our findings correspond comparatively well with the observations of the above authors. High uptake of ^18^FDG in the heart and brain are in accordance with the well known high rate of energetic metabolism in these organs. Remarkably high accumulation of ^18^FDG in Harderian glands found in the present study was observed also by Fukuyama *et al*. (1998), however this phenomenon has not been fully explained yet.

The different profiles of uptake of ^11^C-MET and ^18^FDG found in the present study may undoubtedly be linked to their different metabolism pathways in different organs. This difference might be advantageous in diagnosis of suspected neoplastic processes, brain ischemia, hypoxia, etc. Particularly the low basal accumulation of ^11^C-MET might allow to identify any pathology in this tissue comprising increase of proteosynthesis.
